# Detecting target species: with how many samples?

**DOI:** 10.1098/rsos.220046

**Published:** 2022-08-10

**Authors:** Rosario Delgado

**Affiliations:** Department of Mathematics, Universitat Autònoma de Barcelona, Campus de la UAB, Cerdanyola del Vallès 08193, Spain

**Keywords:** sampling, Poisson, Negative Binomial, detection error, target species

## Abstract

The detection of target species is of paramount importance in ecological studies, with implications for environmental management and natural resource conservation planning. This is usually done by sampling the area: the species is detected if the presence of at least one individual is detected in the samples. Green & Young (Green & Young 1993 Sampling to detectrare species. *Ecol. Appl*. **3**, 351–356. (doi:10.2307/1941837) introduce two models to determine the minimum number of samples *n* to ensure that the probability of failing to detect the species from them, if the species is actually present in the area, does not exceed a fixed threshold: based on the Poisson and the Negative Binomial distributions. We generalize them to two scenarios, one considering the area size *N* to be finite, and the other allowing detectability errors, with probability *δ*. The results in Green & Young are recovered by taking *N* → ∞ and *δ* = 0. Not taking into consideration the finite size of the area, if known, leads to an overestimation of *n*, which is vital to avoid if sampling is expensive or difficult, while assuming that there are no detectability errors, if they really exist, produces an undesirable bias. Our approximation manages to skirt both problems, for the Poisson and the Negative Binomial.

## 1. Introduction

The study of different methods to determine the presence (or absence) of a target species in an area of concern is currently a topic of active research, which has one of its strongholds in the paper by Green & Young [1]. The procedure introduced by the authors for sampling for rare species has been integrated into different monitoring protocols and field studies that have become popular (see, for example, [2,3]) since for some ecological applications an estimate of the prevalence of the species is needed. Of special relevance is the work of Peterson *et al.* [4], within the Western Division of the American Fisheries Society, who developed a protocol to estimate the probability of the presence of the bull trout (*Salvelinus confluentus*) in individual patches (habitat units), which is an endangered species of those considered in the federal Endangered Species Act in the Pacific Northwest. Models based on empirical studies, as is the case here, allow studies to assess sampling efficiency based on habitat characteristics (species abundance distribution (SAD)). For example, in [5] the authors have studied the effect of (under-)sampling as attenuation of the SAD, and how the sampling bias is induced to the SAD by random sampling.

The procedure introduced in [1] has also been used, and shows its usefulness, in the early detection of pest invasions and diseases, which is of paramount importance for the successful management of the possible responses such as containment or eradication, implementing surveillance traps to maximize the probability of detection and minimize economic costs. As an example, in a recent paper [6], the authors model seasonal population dynamics to identify which days of the year are most appropriate for trapping exotic fruit flies (*Diptera: Tephritidae*), getting New Zealand authorities to change the seasonal fruit fly trapping calendar accordingly. The approach followed, based on that of Green & Young [1], is applicable to any invasive species with seasonal variation in surveillance effectiveness. One more example: the authors of Yackel Adams *et al.* [7] introduce a Poisson-based model application to report how long to look to infer the absence of an incipient population of brown tree snakes (*Boiga irregularis*), and claim that their approach applies to other invasive species.

In ecology, it is common to manage count data related to the number of individuals of the target species present per spatial unit. Green & Young [1] considered that since the distribution of a rare species is sparse, it can be assumed that it follows a Poisson distribution. This distribution has also been considered in other fields of biology, such as microbiology, where it has been used, for example, as a model for the total number of viable microbial cells (and clumps) in seeded dilutions, when the organisms have been subjected to some form of sublethal treatment such as freezing, mild heating or disinfectants [8, p. 65]. Since the Poisson distribution with parameter *λ* > 0 has the same expectation and variance, which match the parameter, it is useful as a model in the absence of *overdispersion*. However, when this phenomenon is observed, that is, when the variance is significantly greater than the mean of the distribution of the number of individuals, as in the example borrowed from microbiology when some fraction of the organisms has greater intrinsic resistance or has received a less severe treatment than other cells (see examples 4.3 and 4.4 in [8]), other probability distributions can be used for modelling instead. Among them, the Negative Binomial stands out and it is the one that will be considered in this work.

Count data appearing in ecology and other fields often exhibit simultaneously *overdispersion* and a feature known as *zero inflation*, meaning that an excess of zero values is observed compared to what is expected from the Poisson distribution. Both phenomena are related since *zero inflation* contributes to an increase in the variance of the data, thus producing *overdispersion*. Taking both into account is essential to avoid bias in the construction of ecological statistical models (see [9]). As the Negative Binomial distribution can, at the same time, serve as a model in the presence of these two phenomena, using it we can ‘kill two birds with one stone’. Therefore, we propose the Negative Binomial as a model when *overdispersion* and/or *zero inflation* are present, although otherwise we propose to continue using the classical Poisson distribution.

In this context, the subject under study in this work is the minimum number of samples necessary to take from an area or habitat of interest, say *n*, to ensure that the probability of failing to detect the species in the samples (that is, the probability of not capturing any individual of the species in any of the samples, in case there is no error in detection) if the species is actually present in the area, is at most *β* (*β* ∈ (0, 1), small). Usually *quadrats* are used as samples to collect data and measure biodiversity, being frames traditionally square or rectangular in shape, ranging in size from 1 to 20 m^2^, depending on the habitat being surveyed (https://tools.mheducation.ca/web_resources/sch/ON_Sci_9_Unit1_Sec31.pdf). Without loss of generality, we can assume that any of the samples has an area equal to one unit area. We also assume that the sampling is unbiased, that is, the samples are taken at random from the area. If this were not the case because the samples were taken in a clearly biased way, for logistical or opportunistic reasons, for example, the models we use would no longer be valid and would have to be modified accordingly.

We initially consider the scenario in which the detection of the species in a sample is made without error, that is, we assume that

*f*_*p*_ (*false positive* rate): the probability of detecting the species if it is indeed not present, and
*f*_*n*_ (*false negative* rate): the probability of not detecting the species if it is present,

are both zero, and that the number of individuals of the species follows a Poisson distribution, both at the sample level and at the area (habitat) level, with a mean of *λ* individuals per spatial unit. In Green & Young [1], the general formula

1.1 $$n=\left\lceil -\frac{1}{\lambda }\log(\beta )\right\rceil$$

(where ⌈x⌉ denotes the function *ceiling* that maps *x* to the smallest integer greater than or equal to *x*) follows in this setting, where log denotes the natural logarithm, that is, the logarithm with basis the number *e*. A formula as useful, simple and elegant as (1.1) could not fail to be widely adopted by the scientific community in general, and applied to different fields by research involved in ecology, microbiology and environmental studies (see [4, p. 4], for example).

**Figure 1.**
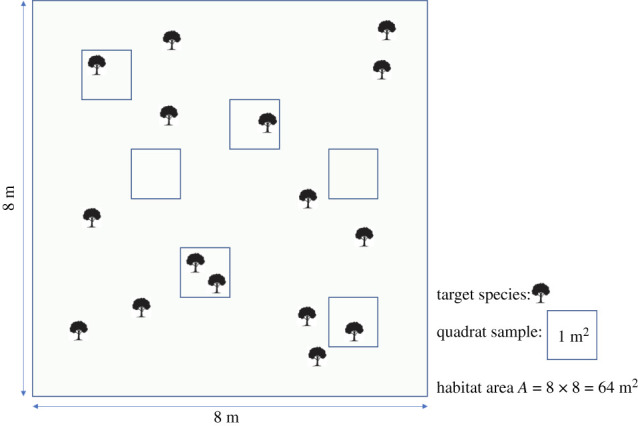
Example of an area *A* of size 64 m^2^, from which six *quadrats* of 1 m^2^ have been chosen at random. Then, *N* = 64.

However, it is possible that the size (surface) of the area of interest, necessarily finite, is known, a situation that the formula (1.1) does not contemplate. As mentioned before, samples are generally *quadrats* of fixed size. Then the number of *quadrats* in the area, say *N*, can be obtained by dividing the size of the area by the size of the *quadrats* (see figure 1 for a visual illustration). Although for obvious reasons it is usually not possible for all of them to have the same shape, this does not invalidate the determination of *N*.

On the other hand, (1.1) also does not contemplate the possibility of error in detecting the presence of individuals of a species in a sample (that is, a positive *false negative* rate, *f*_*n*_ > 0), which is a real threat when the species is rare, that is, its population is scarce, and individuals are difficult to observe, or in situations of insufficient sampling effort. Surveys are known to often miss species present in a sample, even sessile species (see [10] and references therein). The problem is especially serious in ecological studies, where the presence/absence of a species is related to habitat and environmental variables to build habitat-based models [11]. And the same happens with the dynamic models of metapopulations, which predict some ecological processes such as extinction, from the presence/absence data (see [12]), or with models of abundance/occupancy relationships, which are of great interest in metapopulations in biology and in macroecology (see [13] for a closed population, assuming occupancy status does not change during the sampling period, and two generalizations: for open populations in [14], and for non-zero *false positive* rate in [15]). It is also typical of ELISA (enzyme-linked immunosorbent assay), which is a multiwell plate-based immunoassay for the detection of analytes at relatively low cost, whose *sensitivity* (1 − *f*_*n*_) and *specificity* (1 − *f*_*p*_) are usually high but do not reach the maximum value corresponding to the absence of error. Despite this, while the frequency with which errors occur is unknown and will likely vary based on individual experience, false positive errors (recorded occurrences of missing species) are rarely estimated in ecological studies, while the opposite *f*_*n*_ > 0 inevitably occurs in most situations, leading to underestimation occupancy.

With this in mind, our goal in this paper is to present generalizations of the formula (1.1) to two scenarios:

— *Scenario 1.* When the size of the entire area (number of separate samples it contains) is known, say *N*. In this way, we avoid the overestimation in the number of samples *n*, which is the consequence of a simplification in the derivation of (1.1) in the section *Derivation of power formulae* [1], where the authors implicitly assume that the size of the area is infinite (that is, so large that, for practical purposes, it can be considered as such). As the formula (1.1) overestimates the number of samples to be taken, the alternative formula (2.1) is a better alternative as it gives the tightest valor of *n*, if *N* is known. In this paper, we present (2.1) and prove it, in the absence of non-detection error, and from it, (1.1) is the limit case when *N* → ∞.
— *Scenario 2.* Imperfect detectability when the non-detection error is present (*f*_*n*_ ≥ 0). The formula (4.4) takes it into account in the configuration of the infinite area assumption, and generalizes (1.1), which is the particular case when there is no error (*f*_*n*_ = 0).
  Obviously, the two scenarios can occur at the same time, and in this context, we get a generalization of (1.1) both for the case of having finite *N* known and *f*_*n*_ > 0 at the same time, which is (4.1), that incorporates both finiteness of the area size and non-detection errors. While in the first part of the paper (§§2 and 3 and first part of appendix B), we will assume that there is no detection error, that is, *f*_*p*_ = *f*_*n*_ = 0, in §4 and in the second part of appendix B, we assume that *f*_*p*_ = 0 but *f*_*n*_ ≥ 0, that is, there is (possibly) non-detection error in the samples. There, *f*_*n*_ is denoted by *δ* to lighten the notation somewhat.

Moreover, following [1], we consider two different models (in each scenario):

— *model A:* corresponding to the (non-overdispersed) Poisson distribution,
— *model B:* with the Negative Binomial distribution, incorporating both *overdispersion* and *zero inflation*, although we will not consider other models developed specifically for the latter.

The formulae mentioned above correspond to model A, while those corresponding to model B are the generalizations of formula (5.3) that we present in §5. The formula (5.3) appears in [1] as the counterpart to (1.1) when model A is replaced by model B.

In addition, we verified the validity of the models in an experimental phase using Monte Carlo simulation, to approximate the probability of not detecting the presence of the species in the area from the obtained number of samples, *n*, and verifying that, indeed, it is of the order of *β*.

The organization of the paper is as follows: in §2, we consider the scenario 1 and introduce the model A (Poisson distribution) when the detection error has zero probability, if the study area has a finite size. We compare the formula obtained for *n* with that of Green & Young [1], corresponding to an infinite area, in §3. The §4 delves into the adaptation of the Poisson model to scenario 2, considering that the probability of detection error is not zero. The model B (Negative Binomial) is treated in §5. The paper ends with a few words of discussion and conclusion in §6, while in appendix A we prove some technical results, and the topic at hand in appendix B is how to estimate the parameter (mean) of the Poisson distribution (model A).

## 2. Known size of the area (scenario 1): deriving the number of samples with the Poisson model (model A)

We assume that we have a habitat or area, say A, divided into *N* ≥ 1 small areas or samples with the same surface, which we assume without loss of generality to be equal to one unit. We consider that *N*, which is the surface of *A*, is known (a different situation than [1], where it is implicitly assumed that it can be taken as infinity). Denote by *Y* the number of individuals of the species in the entire area *A*, and by *X*_*i*_ the number of individuals of the species in the *i*th sample, *i* = 1, …, *N*. Then, $Y=\sum_{i=1}^{N}X_{i}$. We assume that the random variables *X*_1_, …, *X*_*N*_ are independent, all with the same distribution, a Poisson with parameter (mean value) *λ* > 0, insensitive to the position and shape of the sample. This is what we call model A, and we are in the scenario 1.

Denote by *X* a random variable with Poisson distribution with parameter *λ*, *X* ∼ Pois(*λ*). For the moment we assume that *λ* is known (we will return to this topic in appendix B). Since *Y* is the sum of *N* independent Poisson variables with the same parameter *λ*, *Y* follows a Poisson distribution with parameter the sum of parameters, that is, with parameter *λN* ( ≥*λ* > 0), *Y* ∼ Pois(*λN*). With *β* introduced earlier as the upper bound for the probability of not capturing any individuals of the species in any of the samples if the species is actually present in the area, established by the research team, we can now derive the formula for the minimum number of samples, *n*, such that this probability is, in fact, less than or equal to *β*.

**Theorem 2.1.**

*In scenario 1 and with model A, the minimum number of samples *n* to extract from area A such that the probability of not capturing any individual of the species in any of the samples if the species is actually present in the area, less than or equal to *β*, is*

2.1 $$n=\left\lceil -\frac{1}{\lambda }\log(\beta +(1-\beta )\;e^{-\lambda N})\right\rceil >0.$$

**Proof.**

First, we see that the value of *n* given by the formula (2.1) is positive. Indeed, since *λ* > 0, we have to check that *β* + (1 − *β*) e^−*λN*^ ∈ (0, 1) (since then, its logarithm is a negative number). In fact, since *β* ∈ (0, 1), the positiveness of the exponential ensures that this quantity is strictly positive. Besides,

$$\beta +(1-\beta )\;e^{-\lambda N}<1⟺e^{-\lambda N}<\frac{1-\beta }{1-\beta }=1,$$

which holds due to the fact that −*λN* < 0. Second, we check that *n* ≤ *N*, which holds since

$$\log(\beta +(1-\beta )\;e^{-\lambda N})>-\lambda N⟺\beta +(1-\beta )\;e^{-\lambda N}>e^{-\lambda N}⟺\beta (1-e^{-\lambda N})>0$$

(thus, −(1/*λ*)log (*β* + (1 − *β*) e^−*λN*^) < *N* and then *n* ≤ *N*).

Now we deduce the expression (2.1). For any *n* ≥ 1 (*n* ≤ *N*), we draw *n* samples at random from the area *A*, and we can assume without loss of generality that they are the first, say samples 1, …, *n*. Then, not capturing any individual of the species in any of the *n* samples is equivalent to saying that $\sum_{i=1}^{n}X_{i}=0$. With this in mind, the goal is to find the minimum number *n* such that

2.2 $$P\left(\sum_{i=1}^{n}X_{i}=0\;/\;Y>0\right)\leq \beta .$$

We determine the minimum integer *n* that verifies (2.2) as follows:

2.3 $$\begin{array}{rl} P\left(\sum_{i=1}^{n}X_{i}=0\;/\;Y>0\right) & =\frac{P\left(\sum_{i=1}^{n}X_{i}=0⋂Y>0\right)}{P(Y>0)}=\frac{P\left(\sum_{i=1}^{n}X_{i}=0⋂\sum_{i=n+1}^{N}X_{i}>0\right)}{P(Y>0)}\\ & =\frac{P\left(\sum_{i=1}^{n}X_{i}=0\right)P\left(\sum_{i=n+1}^{N}X_{i}>0\right)}{P(Y>0)} \end{array}$$

where we have used that $\sum_{i=1}^{n}X_{i}$ and $\sum_{i=n+1}^{N}X_{i}$ are independent random variables, which is a consequence of the assumption of independence of *X*_1_, …, *X*_*N*_. Then, since they are sums of independent random variables with Poisson distribution with the same parameter *λ*, they have distributions Pois(*λn*) and Pois(*λ*(*N* − *n*)), respectively. Then, by (2.3) we can write

2.4 $$P\left(\sum_{i=1}^{n}X_{i}=0\;/\;Y>0\right)=\frac{e^{-\lambda n}(1-e^{-\lambda (N-n)})}{1-e^{-\lambda N}}=\frac{e^{-\lambda n}-e^{-\lambda N}}{1-e^{-\lambda N}}.$$

Finally, we can isolate *n* from (2.4) and (2.2). In fact, by (2.4),

$$(2.2)⟺e^{-\lambda n}-e^{-\lambda N}\leq \beta (1-e^{-\lambda N})⟺e^{-\lambda n}\leq \beta +(1-\beta )\;e^{-\lambda N}$$

and taking the natural logarithm, which is an increasing function, on the two sides of the inequality:

$$(2.2)⟺-\lambda n\leq \log(\beta +(1-\beta )\;e^{-\lambda N})⟺n\geq -\frac{1}{\lambda }\log(\beta +(1-\beta )\;e^{-\lambda N}).$$

Then, the minimum integer value of *n* is given by this expression if it is an integer, or the next higher positive integer if it is not, finishing the proof. ▪

Green & Young [1, p. 352] define *β* (and here we quote the authors) as ‘the probability of allocating *n* quadrats and failing to collect a species *that is actually present in that habitat* and has some mean density *m*’ (note that with our notation, *m* = *λ*). So, *β* is clearly the probability of not detecting *conditional* on the presence of the species in the habitat. The problem is that these authors implicitly assume that *P*(*Y* > 0) = 1, which is contradictory to the Poisson model, for which *P*(*Y* > 0) = 1 − e^−*λN*^, except if we are in the limit situation where fixed *λ*, *N* → +∞. Indeed, in [1] the expression (2.2) is replaced by

$$P\left(\sum_{i=1}^{n}X_{i}=0\right)\leq \beta ,$$

with $\sum_{i=1}^{n}X_{i}∼\mathrm{Pois}(\lambda n)$, which is equivalent to e^−*λn*^ ≤ *β*, that translates into (1.1).

The problem of estimating *λ* from the available information (data) is covered in appendix B.

## 3. Comparing with the formula (1.1)

In the following proposition, whose proof is in appendix A, we show that, indeed, the value of *n* given by (2.1) is not greater than that given by (1.1), although they tend to match when *N* → ∞. What is more, it gives an upper bound on the difference between (1.1) and (2.1), stating exactly what that difference is for *N* large enough. In the experimental simulation phase at the end of this section, we will see that if *N* is known, (2.1) is a refinement (providing a tighter value) of (1.1), which is, therefore, an overestimate (see examples 1 and 2 in table 2).

**Proposition 3.1.**

*For any* *β* ∈ (0, 1), *λ* > 0 *and* *N* ≥ 1, *we have that*

(a) 0 ≤ (1.1)–(2.1)$\;\leq \lceil (1/\lambda )\log(1+(\left(1-\beta \right)/\beta )\;e^{-\lambda N})\rceil$,
(b) $\lim_{N\to +\infty }$(2.1) = (1.1), *and*
(c) *for* *N* *large enough*, (1.1)–(2.1)$\;=\lceil (1/\lambda )\log(1+(\left(1-\beta \right)/\beta )\;e^{-\lambda N})\rceil -1\geq 0$.

### 3.1. Examples

As example 1, we consider the particular case where *β* = 0.05 and *λ* = 0.001. Applying (1.1) we obtain *n* = 2996. Therefore, to record the improvement in determining the minimum number of samples *n* needed to extract from the area A, such that the probability of detecting the presence of the species, if it is actually present in area, is not greater than *β*, for different values of *N* using formula (2.1), we start with *N* = 3000 (≥2996). The recorded values appear in table 1, along with another example where *β* remains unchanged but *λ* = 0.01, yielding (1.1)=300.

**Table 1.** Two examples of how the overestimation of *n* using the formula (1.1), with respect to the formula (2.1), decreases as *N* increases. In both cases, the maximum overstatement (achieved with the minimum *N*) is greater than 28% (100×666/2330=28.58% and 100×67/233=28.76%).

example 1: β=0.05,λ=0.001,(1.1)=2996			example 2: β=0.05, λ=0.01,(1.1)=300		
*N*	*n* given by (2.1)	(1.1)–(2.1)	*N*	*n* given by (2.1)	(1.1)–(2.1)
3000	2330	666	300	233	67
3500	2543	453	350	255	45
4000	2698	298	400	270	30
4500	2805	191	450	281	19
5000	2876	120	500	288	12
5500	2921	75	550	293	7
6000	2950	46	600	295	5
6500	2968	28	650	297	3
7000	2979	17	700	298	2
7500	2986	10	750	299	1
8000	2990	6	800	299	1
8500	2992	4	850	300	0
9000	2994	2	900	300	0
9500	2995	1	950	300	0
10 000	2995	1	1000	300	0
10 500	2996	0	1050	300	0
11 000	2996	0	1100	300	0

In example 2, we start with *N* = 300. As we can see from these examples, if obtaining samples is expensive or difficult, the savings on samples by using (2.1) instead of (1.1), if *N* is known, can really be worth the effort.

### 3.2. Experimental simulation phase

We can carry out a simulation experiment in which, fixed *β* and *λ*, for the value of *n* given by (2.1), we approximate the probability *p* of not detecting the presence of the species in area *A* from the *n* samples, using a Monte Carlo method (by the Law of the Large Numbers), and verify that indeed, this probability is very close to *β*. If, instead, *n* is given by the formula (1.1), the probability obtained by simulation is clearly much lower, showing the overestimation of *n* in [1]. Naturally, *N* and *λ* fixed, the higher the number of samples *n*, the lower the probability *p*, as the examples in table 2.

**Table 2.** *K* = 10^7^ iterations of the algorithm 1 for some values of *N* in the two examples of table 1, both using (2.1) and (1.1) to determine *n*. *p* = Probability of not detecting the presence of the species in area *A*, if present, from the *n* samples, which must be approximately equal to *β*, ideally not greater, although the simulation procedure may lead to a result that (narrowly) violates this constraint.

example 1: *β* = 0.05, *λ* = 0.001			example 2: *β* = 0.05, *λ* = 0.01		
*N*	*n*	*p*	*N*	*n*	*p*
3000	2330 (2.1)	0.05003096	300	233 (2.1)	0.05001881
	2996 (1.1)	0.00021405		300 (1.1)	0.00000000
4000	2698 (2.1)	0.04991710	400	270 (2.1)	0.04977368
	2996 (1.1)	0.03213956		300 (1.1)	0.03198875
5000	2876 (2.1)	0.04994049	500	288 (2.1)	0.04973971
	2996 (1.1)	0.04360176		300 (1.1)	0.04333699

Therefore, it is about looking for a trade-off between *n* and *p*, taking into account that when *n* improves (decreases), *p* worsens (increases) and vice versa. The compromise solution that we have adopted has been to establish an upper bound for *p*, *β*, to determine the smallest number of samples *n* that guarantees that *p* does not exceed this bound. In doing so, with (2.1) we find a very tight value of *n*, which is, however, overestimated when (1.1) is used instead. This is precisely the leitmotiv of this work. We have implemented the algorithm that performs the simulation (see algorithm 1) using the R programming language [16].


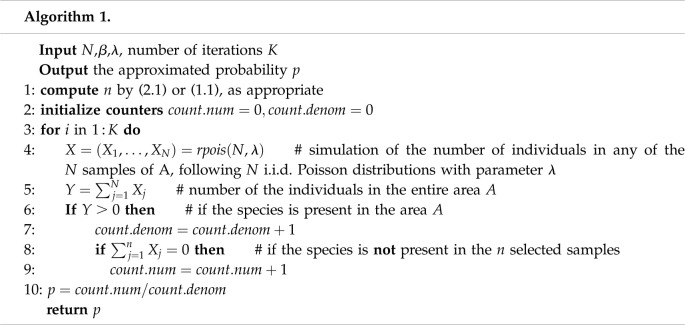


With the examples in table 1, with *K* = 10^7^ iterations, we obtain the results in table 2 by applying algorithm 1 to *n* obtained both from (2.1) and from (1.1).

## 4. What if detectability is imperfect? (scenario 2 with model A)

We can introduce the non-detection error (scenario 2) and study its effect on the previous formulae (model A). This is the error of not detecting the presence of an individual of the species in a sample in which it is actually found. We denote by *δ* ∈ [0, 1) its probability, i.e. the *false negative* rate *f*_*n*_ (we use *δ* instead of *f*_*n*_ to lighten the notation a bit), and we assume that this error occurs (or does not) independently for the different individuals of the species present in the samples. The opposite error (*false positive*), which corresponds to recording the presence of a species in a sample when it is not there, is assumed never to occur. We can prove the following result, similar to theorem 2.1, where we observe the effect of detectability error in determining the minimum number of samples.

**Theorem 4.1.**

*In scenario 2 and with model A, with detectability error* *δ* ∈ [0, 1), *the minimum number of samples* *n* *to extract from area A such that the probability of not capturing any individual of the species in any of the samples if the species is actually present in the area, less than or equal to* *β*, *is*

4.1 $$n=\left\lceil -\frac{1}{\lambda (1-\delta )}\log(\beta +(1-\beta )\;e^{-\lambda N})\right\rceil >0.$$

**Proof.**

The presence of the non-detection error affects expression (2.2), which now becomes:

4.2 $$P\left(\sum_{i=1}^{n}X_{i}=0⋃(\sum_{i=1}^{n}X_{i}>0⋂\text{no detect})\;/\;Y>0\right)\leq \beta$$

and then, similarly to (2.3) and (2.4), we can expand the probability on (4.2) in this way:

4.3 $$\begin{array}{rl} & P\left(\sum_{i=1}^{n}X_{i}=0⋃(\sum_{i=1}^{n}X_{i}>0⋂\text{no detect})\;/\;Y>0\right)\\ & \;=\frac{P\left(\sum_{i=1}^{n}X_{i}=0⋂Y>0\right)+P\left(\sum_{i=1}^{n}X_{i}>0⋂\text{no detect}⋂Y>0\right)}{P(Y>0)}\\ & \;=\frac{P\left(\sum_{i=1}^{n}X_{i}=0⋂\sum_{i=n+1}^{N}X_{i}>0\right)+P\left(\sum_{i=1}^{n}X_{i}>0⋂\text{no detect}\right)}{P(Y>0)}\\ & \;=\frac{e^{-\lambda n}(1-e^{-\lambda (N-n)})+(*)}{1-e^{-\lambda N}} \end{array}$$

with

$$\begin{array}{rl} \left(*\right) & =P\left(\sum_{i=1}^{n}X_{i}>0⋂\text{no detect}\right)=\sum_{\;j=1}^{\infty }P\left(\sum_{i=1}^{n}X_{i}=j⋂\text{no detect}\right)\\ & =\sum_{\;j=1}^{\infty }P\left(\text{no detect }\;/\;\sum_{i=1}^{n}X_{i}=j\right)P\left(\sum_{i=1}^{n}X_{i}=j\right)=\sum_{\;j=1}^{\infty }\delta^{j}\;e^{-\lambda n}\frac{{\left(\lambda n\right)}^{j}}{j!} \end{array}$$

where we have used that $\sum_{i=1}^{n}X_{i}∼\mathrm{Pois}(\lambda n)$. Then,

$$\left(*)=e^{-\lambda n}\sum_{\;j=1}^{\infty }\frac{{\left(\lambda n\delta \right)}^{j}}{j!}=e^{-\lambda n}(e^{\lambda n\delta }-1\right)$$

Finally, substituting (*) in (4.3) we have

$$\begin{array}{rl} & P\left(\sum_{i=1}^{n}X_{i}=0⋃(\sum_{i=1}^{n}X_{i}>0⋂\text{no detect})\;/\;Y>0\right)\\ & \;=\frac{e^{-\lambda n}(1-e^{-\lambda (N-n)})+e^{-\lambda n}(e^{\lambda n\delta }-1)}{1-e^{-\lambda N}}\\ & \;=\frac{e^{-\lambda n}(e^{\lambda n\delta }-e^{-\lambda (N-n)})}{1-e^{-\lambda N}}=\frac{e^{-\lambda n(1-\delta )}-e^{-\lambda N}}{1-e^{-\lambda N}} \end{array}$$

and imposing (4.2), we get

$$n\geq -\frac{1}{\lambda (1-\delta )}\log(\beta +(1-\beta )\;e^{-\lambda N}),$$

ending the proof. ▪

The estimation of *λ* for model A in scenario 2 from available data is also considered in appendix B.

If *δ* = 0 (*false negative* rate *f*_*n*_ equals zero), the formulae (4.1) and (2.1) coincide. In the limit, when *δ* → 1, the formula (4.1) converges to ∞, which is reasonable since it corresponds to the unlikely situation in which the probability of non-detection is 1, that is, the probability of detecting the presence of the species in a sample where it is present, is zero. In the intermediate cases, (4.1)≥(2.1), which is logical, since the existence of non-detection error leads to a larger number of samples. In other words, the minimum number of samples to be taken from the area can be substantially different depending on whether detectability error is taken into account and, in the first case, depending on the magnitude of the error.

If we take the limit as *N* → ∞ in (4.1) we get the equivalent of (1.1) in the scenario of non-detection error with probability *δ*, which is the following expression:

4.4 $$n=\left\lceil -\frac{\log(\beta )}{\lambda (1-\delta )}\right\rceil$$

and if *δ* = 0, (4.4) and (1.1) match; in the limit, when *δ* → 1, the formula (4.4) converges to +∞; and in the intermediate cases, (4.4)≥(1.1).

Analogous to proposition 3.1, we obtain the following result that we state without proof.

**Proposition 4.2.**

*For any* *β* ∈ (0, 1), *λ* > 0, *N* ≥ 1 and *δ* ∈ [0, 1), *we have that*

(a) $0\leq (4.4)-(4.1)\leq \lceil (1/\left(\lambda \;(1-\delta )\right))\log(1+(\left(1-\beta \right)/\beta )\;e^{-\lambda N})\rceil$,
(b) $\lim_{N\to +\infty }(4.1)=(4.4)$, *and*
(c) *for* *N* *large enough*, $(4.4)-(4.1)=\lceil (1/\left(\lambda \;(1-\delta )\right))\log(1+(\left(1-\beta \right)/\beta )\;e^{-\lambda N})\rceil -1\geq 0$.

In table 3, we record the approximation for the probability of not detecting the presence of the species in the area, if it is present, from the *n* samples, in two examples: example 3, with *false negative* rate *δ* ranging from 0 (no detection error) to 0.1, and *N* = 3500, and example 4, with *δ* = 0.1 to 0.5 and *N* = 6000. In both examples, *β* = 0.05 and *λ* = 0.001. The estimated values of *p* have been obtained by simulation using the algorithm 2, implemented with the programming language R. Note that with *δ* = 0, the algorithms 2 and 1 are, in fact, the same.

**Table 3.** *K* = 10^7^ iterations of algorithm 2 for examples 3 and 4. *p* = Probability of not detecting the presence of the species in area *A*, if present, from the *n* samples, with *δ* the probability of not detecting any individual.

example 3: *N* = 3500, *β* = 0.05, *λ* = 0.001				
*δ*	*n* (4.1)	*p*	*n* (4.4)	*p*
0.0000	2543	0.04980696	2996	0.02044645
0.0001	2543	0.05005280	2997	0.02037177
0.0005	2544	0.04998135	2998	0.02044940
0.0010	2545	0.04998002	2999	0.02043230
0.0050	2556	0.04984517	3011	0.02044562
0.0100	2568	0.04991535	3026	0.02041467
0.0500	2677	0.04993774	3154	0.02035112
0.1000	2825	0.05009235	3329	0.02046655
example 4: *N* = 6000, *β* = 0.05, *λ* = 0.001				
*δ*	*n* (4.1)	*p*	*n* (4.4)	*p*
0.1	3278	0.04996806	3329	0.04766190
0.2	3688	0.04992643	3745	0.04749297
0.3	4214	0.05003753	4280	0.04770510
0.4	4917	0.04991602	4993	0.04761596
0.5	5900	0.04993702	5992	0.04759538


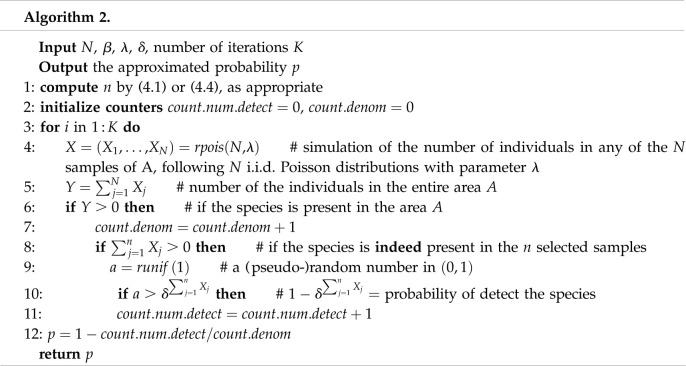


In the examples in table 3, we observe two phenomena in addition to the expected fact that the larger the value of *N*, the smaller the difference between the two models: (i) that using (4.4) instead of (4.1) to determine the number of samples *n* gives an overestimate, since the approximate probability by simulation is unnecessarily much smaller than *β* = 0.05 and (ii) that the values obtained for *p* remain stable when *δ* increases. This seems quite common sense because the formulae (4.1) and (4.4) have been obtained precisely to determine the minimum value of *n* that ensures, with each model, that the probability *p* of not detecting the presence of the species in area *A* with the *n* samples is less than or equal to *β*, considering the presence of the false negative error *δ*. Since the second model is less fit than the first, it effectively leads to an overestimation of *n*, which translates into a lower value of *p*, clearly below *β*.

## 5. Oversampling and zero inflation. Model B: Negative Binomial distribution

As explained in the introduction (§1), in the presence of *oversampling* and/or *zero inflation* phenomena, the Poisson distribution (model A) is no longer a suitable model for counting individuals per unit area. Instead, we consider the Negative Binomial distribution (model B). We first consider the scenario 1 (finite size *N* of the area of interest), and that there are no detection errors (*δ* = 0).

Analogously to §2, if we denote by *Y* the number of individuals of the species in the entire area *A*, and by *X*_1_, …, *X*_*N*_ the number of individuals of the species in each of the *N* samples that make up A, now we assume that these random variables are independent, all with Negative Binomial distribution with parameters *r* (a positive integer) and *p* ∈ (0, 1). Let *X* be a counting random variable with distribution NB(*r*, *p*). So, for any *k* ≥ 0,

$$P(X=k)=\left(\begin{array}{c} k+r-1\\ r-1 \end{array}\right)p^{k}{\left(1-p\right)}^{r}$$

and its expectation and variance are, respectively, *E*(*X*) = *rp*/(1 − *p)* and Var(*X*) = *rp*/(1 − *p*)^2^. Note that since *p* ∈ (0, 1), we have that Var(*X*) > *E*(*X*) (*overdispersion*), unlike what happens in model A, with the Poisson distribution, in which case *E*(*X*) = Var(*X*) = *λ*. If we denote by *λ* the expectation of *X* ∼ NB(*r*, *p*), that is, *λ* = *rp*/(1 − *p)*, then *p* = *λ*/(*r* + *λ*) and we have that Var(*X*) = *λ*(1 + (*λ*/*r*)) > *λ*, and

$$\frac{\mathrm{Var}(X)}{E(X)}=\frac{\lambda (1+\lambda /r)}{\lambda }=1+\frac{\lambda }{r}>1$$

is known as the *dispersion index* [17], which is 1 for the Poisson distribution. As stated in §1, the Negative Binomial distribution not only captures the phenomenon of *overdispersion*, but also that of *zero inflation* since *P*(*X* = 0) = (1 − *p*)^*r*^ = (1 − *λ*/(*r* + *λ*))^*r*^ is greater than the mass given at zero by Pois(*λ*), which is e^−*λ*^. In fact, what happens is that

5.1 $$\lim_{r\to \infty }{\left(1-\frac{\lambda }{r+\lambda }\right)}^{r}=\lim_{r\to \infty }{\left(\frac{1}{1+\lambda /r}\right)}^{r}=e^{-\lambda }$$

being a decreasing sequence, and in the sense of the limit of the distributions,

$$\lim_{r\to \infty }\mathrm{NB}(r,p)=\lim_{r\to \infty }\mathrm{NB}\left(r,\frac{\lambda }{r+\lambda }\right)=\mathrm{Pois}(\lambda ).$$

As the sum of independent random variables with distribution NB(*r*, *p*) is a distribution of the same type with first parameter the sum, we have that *Y* ∼ NB(*rN*, *p*). Recall that *β* is the upper bound of the probability of not capturing any individual of the species in any of the samples, if the species is actually present in the area. Then, we can obtain the expression for the minimum number of samples, *n*, such that this probability is less than or equal to *β*, in the following theorem.

**Theorem 5.1.**

*In scenario 1 and with model B, the minimum number of samples* *n* *to extract from area A such that the probability of not capturing any individual of the species in any of the samples if the species is actually present in the area, is less than or equal to* *β*, *is*

5.2 $$n=\left\lceil \frac{-1}{r}\frac{\log(\beta +(1-\beta ){\left(1/\left(1+\lambda /r\right)\right)}^{rN})}{\log(1+\lambda /r)}\right\rceil >0$$

**Proof.**

The proof is similar to that of theorem 2.1 considering that if *X* ∼ NB(*r*, *p*) then

$$P(X=0)={\left(1-p\right)}^{r}={\left(1-\frac{\lambda }{r+\lambda }\right)}^{r}={\left(\frac{1}{1+\lambda /r}\right)}^{r}$$

where *λ* = *rp*/(1 − *p*). And with respect to *Y* ∼ NB(*rN*, *p*), we have that

$$P(Y=0)={\left(1-p\right)}^{rN}={\left(1-\frac{\lambda_{Y}}{rN+\lambda_{Y}}\right)}^{rN}={\left(\frac{1}{1+\lambda /r}\right)}^{rN}$$

(where *λ*_*Y*_ denotes the expectation of the variable *Y*, *λ*_*Y*_ = *Nrp*/(1 − *p*) = *Nλ*). Therefore, since $\sum_{i=1}^{n}X_{i}$ and $\sum_{i=n+1}^{N}X_{i}$ are independent random variables, with respective distributions NB(*rn*, *p*) and NB(*r*(*N* − *n*), *p*), we have that

$$\begin{array}{rl} P\left(\sum_{i=1}^{n}X_{i}=0\;/\;Y>0\right) & =\frac{{\left(1/\left(1+\lambda /r\right)\right)}^{r\;n}\;(1-{\left(1/\left(1+\lambda /r\right)\right)}^{r\;(N-n)})}{1-{\left(1/\left(1+\lambda /r\right)\right)}^{r\;N}}\\ & =\frac{{\left(1/\left(1+\lambda /r\right)\right)}^{r\;n}-{\left(1/\left(1+\lambda /r\right)\right)}^{r\;N}}{1-{\left(1/\left(1+\lambda /r\right)\right)}^{r\;N}} \end{array}$$

and imposing that this probability is less than *β*, we can isolate *n* and obtain

$$n\geq \frac{\log(\beta +(1-\beta ){\left(1/\left(1+\lambda /r\right)\right)}^{rN})}{r\log(1/\left(1+\lambda /r\right))}$$

which ends the proof. ▪

### 5.1. Special situations

#### 5.1.1. Model A as limit of model B when *r* → +∞

Note that if in the formula (5.2) we take the limit as *r* → +∞, by using (5.1), we rediscover the formula (2.1) corresponding to model A with the Poisson distribution.

#### 5.1.2. The limit when *N* → ∞

On the other hand, taking the limit as *N* → ∞ in (5.2) we obtain the expression corresponding to (1.1) with the Negative Binomial model B, which is

5.3 $$n=\left\lceil \frac{-1}{r}\frac{\log(\beta )}{\log(1+\lambda /r)}\right\rceil$$

from which (1.1) can be found again by taking the limit as *r* → +∞. Formula (5.3) matches formula (3) in [1].

#### 5.1.3. Model B in scenario 2 (imperfect detectability)

Recall that *δ* ∈ [0, 1) is the probability of the error corresponding to not detecting the presence of any individual of the species in any sample in which the species is actually found, and that we assume that the error corresponding to recording the presence of a species in a sample never occurs when it is not present. Analogously to theorems 5.1 and 4.1, we obtain that

5.4 $$n=\left\lceil \frac{-1}{r}\frac{\log(\beta +(1-\beta ){\left(1/\left(1+\lambda (1-\delta )/r\right)\right)}^{rN})}{\log(1+\lambda (1-\delta )/r)}\right\rceil >0.$$

If we take the limit as *N* → +∞ we get the formula corresponding to (4.4) for model B:

5.5 $$n=\left\lceil \frac{-1}{r}\frac{\log(\beta )}{\log(1+\lambda (1-\delta )/r)}\right\rceil .$$

### 5.2. Parameter estimation in model B

Note that the parameter pair (*r*, *λ*) is equivalent, though statistically preferred for estimation purposes, to the usual parametrization (*r*, *p*) of the Negative Binomial, and henceforth we will refer to this distribution using the parametrization (*r*, *λ*).

How can the parameters (*r*, *λ*) of the model B be obtained? We randomly take an arbitrary number *n*_0_ of samples from area *A* (the larger, the better estimates we get), and denote by $x_{1},\ldots ,x_{n_{0}}$ the realization of variables $X_{1},\ldots ,X_{n_{0}}$, denoting *the number of individuals of the species in each of the* *n*_0_ *samples*. If *δ* = 0, $x_{1},\ldots ,x_{n_{0}}$ can be observed, and the natural (biased) moment estimators of the parameters are

5.6 $$\text{model B },\delta =0\;:\;\;\hat{\lambda }=\bar{x},\;\hat{r}=\frac{{\left(\bar{x}\right)}^{2}}{s_{x}^{2}-\bar{x}}$$

with the notations

$$\bar{x}=\frac{1}{n_{0}}\sum_{i=1}^{n_{0}}x_{i}\;\text{( the sample mean value) }$$

and

$$s_{x}^{2}=\frac{1}{n_{0}}\sum_{i=1}^{n_{0}}x_{i}^{2}-{\left(\bar{x}\right)}^{2}\;\text{(the sample variance without Bessel's correction) }$$

If *δ* > 0, the number of individuals of the species present in each of the *n*_0_ samples cannot be observed, and instead $x_{1},\ldots ,x_{n_{0}}$ denote *the number of individuals of the species actually detected in any of the* *n*_0_ *samples*. From these observed values we estimate the parameters by

5.7 $$\text{model B },\delta >0\;:\;\;\hat{\lambda }=\frac{\bar{x}}{1-\delta },\;\hat{r}=\frac{{\left(\bar{x}\right)}^{2}}{s_{x}^{2}-\bar{x}}.$$

## 6. Discussion and conclusion

Studying the presence/absence of target species in an area of interest is one of the most important tasks in ecological studies, with implications for the environmental management and planning for the conservation of natural resources. Given the impossibility of carrying out an exhaustive follow-up of the entire study area, a common case in practice, samples of a certain size, which can be assumed to be 1 without loss of generality, are taken to determine from them the presence/absence of the species of interest. Sampling necessarily induces uncertainty in our conclusions and we have used different probabilistic models for this uncertainty, which allow addressing the question of determining the minimum number of samples, *n*, necessary to ensure that the probability of failing to detect the species from them if the species is actually present in the area is at most a fixed (and small) *β* ∈ (0, 1).

Specifically, we use the Poisson model, and its counterpart, the Negative Binomial model, when there is overdispersion and/or zero inflation. These models have been introduced in [1] but implicitly for a horizon in which the size of the area is infinite. In this work, we have adapted the models to incorporate the size of the area (equivalently, the number of non-overlapping samples that could be drawn from it, *N*) and show that not taking this information into account, when it is known, always leads to an overestimate of *n*. If obtaining samples is expensive or difficult, the savings on samples with the approach presented in this paper can really be worth it.

When building models for the occupancy and abundance of wild species in a given habitat, evaluation of the predictive accuracy of the models depends on the reliability of the data. If the data lack reliability because there are individuals of the species that are not detected as such by mistake, that is, if detection error (*false negative*) is present, the models must be adjusted accordingly. In general, the higher the probability of the detection error, the lower the efficiency of the model and the greater the bias in the estimation of the parameters.

Fortunately, ecological models of wildlife habitat based on the presence/absence data that assume detection errors are now quite common. These models assume that if a species is present in a given sample, its presence is not detected with probability (*false negative* rate) *f*_*n*_ = *δ* > 0. And this is of great importance to be aware of the presence of this detection error, and to act accordingly in the construction of the model, since the models that do not take it into account will suffer from bias in the estimation of their parameters [11].

Some of the researchers who have dealt with this type of model accept the presence of this bias and focus on ensuring the usefulness of the model despite detection error. An example is [18], which points out that the methodology of estimating the relative abundance of a species using a machine learning classification algorithm to detect the species in areas where its presence had not been previously confirmed, can be applied to compare the relative abundances between different areas if the detection error is the same in all of them. Others, such as Blasco-Moreno *et al.* [9], follow a different approach, considering the presence of *false zeros* due to observer detection errors (or errors in the experimental design) and suggesting to minimize their presence when performing the experiment, before building a statistical model for the occupancy/abundance of the species of interest, if possible removing them from the dataset before analysis. And still other authors focus on trying to avoid the negative bias in the estimation of parameters derived from the fact that species can go unnoted even though they are present. For example, MacKenzie *et al.* [19] propose a likelihood-based method to estimate site occupancy rates when the probability of detection error is positive, which avoids bias in estimating the proportion of occupied patches when there is error detection.

Our work aligns with the latter: if the detection error *δ* is not taken into consideration in the determination of *n*, it is underestimated, that is, it is estimated with a negative bias. What we have done in this work to avoid this bias is to adapt a model that was suitable when *δ* = 0, to the case in which *δ* > 0, thus avoiding the underestimation of *n*. That is, beyond the convenience of investigating which variables affect the probability of non-detection, as was done in [20], assuming the probability of non-detection *δ* ∈ [0, 1) is known, we provide a version of the formulae obtained when this probability is assumed to be zero, which are *δ* dependent.

The models we propose are a combination of assumptions about (i) the distribution of site occupancy and (ii) the detection probabilities, which seems to provide a useful flexible framework for statisticians and biologists. From a statistician’s point of view, surveying for target species is similar whether the area is small or large, or whether the biological population is rare or abundant. However, studies of a rare species have more complicated logistics, requiring more time and resources, especially if the probability of detection is not vey great. For this reason, models that provide an adjusted estimate of the number of samples from the area of interest are necessary to ensure that the probability of failing to detect a rare species, if it is actually present in the area, is at most a fixed threshold, like the ones we present here, are undeniably useful for applications in ecology such as (i) management of invasive species (due to limited resources, government agencies often give priority to interventions to invasive exotic species), (ii) management of threatened species that, by definition, are rare in the study area, (iii) spatial planning (decisions about which areas will be protected for the conservation of the species) and (iv) biogeography (identification of biodiversity hotspots from species richness studies).

**Figure 2.**
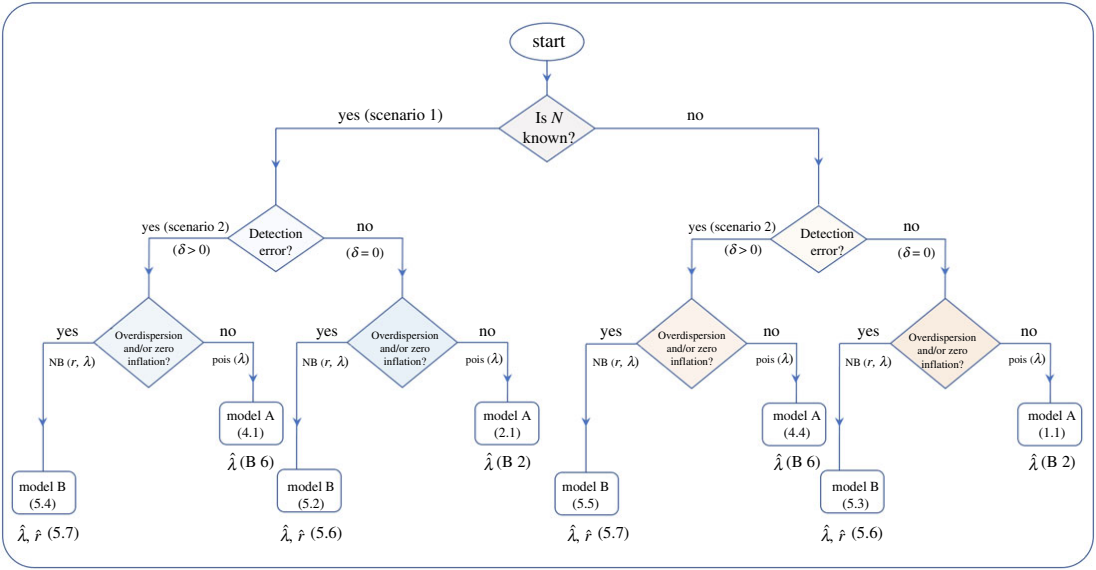
Pipeline of practice to implement the approach to find the minimum number of samples *n* to ensure that the probability of failing in detecting the species from them, if the species is actually present in the area, does not exceed a fixed threshold *β*.

Figure 2 schematically shows the procedure to be followed, from a practical point of view, to determine *n* according to the different scenarios, and indicates the formulae to be used in each situation, which are summarized in table 4. Table 5 provides the estimates of the parameters of both models A and B.

**Table 4.** Summary of formulae to determine *n*. Taking the limit as *r* → +∞ in the formulae corresponding to the Negative Binomial (model B) we obtain those corresponding to the Poisson model (model A).

model A: Poisson distribution Pois(*λ*)		
number of samples *n*	scenario 1. Area size *N*	*N* → +∞
scenario 2*. δ* > 0	$\left\lceil -\frac{1}{\lambda (1-\delta )}\log(\beta +(1-\beta )\;e^{-\lambda N})\right\rceil$ (4.1)	$\left\lceil -\frac{\log(\beta )}{\lambda (1-\delta )}\right\rceil$ (4.4)
*δ* = 0	$\left\lceil -\frac{1}{\lambda }\log(\beta +(1-\beta )\;e^{-\lambda N})\right\rceil$ (2.1)	$\left\lceil -\frac{1}{\lambda }\log(\beta )\right\rceil$ (1.1)
model B: Negative Binomial distribution NB(*r*, *λ*)		
number of samples *n*	scenario 1. Area size *N*	*N* → +∞
scenario 2*. δ* > 0	$\left\lceil \frac{-1}{r}\frac{\log(\beta +(1-\beta ){\left(1/\left(1+(\lambda (1-\delta )/r)\right)\right)}^{r\;N}\;)}{\log(1+(\lambda (1-\delta )/r))}\right\rceil$ (5.4)	$\left\lceil \frac{-1}{r}\;\frac{\log(\beta )}{\log(1+(\lambda (1-\delta )/r))}\right\rceil$ (5.5)
*δ* = 0	$\left\lceil \frac{-1}{r}\frac{\log(\beta +(1-\beta )\;{\left(1/\left(1+(\lambda /r)\right)\right)}^{rN})}{\log(1+(\lambda /r))}\right\rceil$ (5.2)	$\left\lceil -\frac{1}{r}\;\frac{\log(\beta )}{\log(1+(\lambda /r))}\right\rceil$ (5.3)

**Table 5.** Summary of estimates. Model A: *w*_*n*_ is the number of the *n* samples for which we have detected the presence of the species (= the number of them that contain at least one individual, if *δ* = 0). Model B: *x*_1_, …, *x*_*n*_ are the number of individuals of the species that have been detected in any of the samples (= the number of individuals indeed present in any of them, if *δ* = 0). $\bar{x}$ is the sample mean value, $s_{x}^{2}$ is the sample (uncorrected) variance.

	model A: Pois(*λ*)	model B: NB(*r*, *λ*)
scenario 2. *δ* > 0	$\hat{\lambda }=-\frac{\log(1-\hat{\alpha })}{1-\delta }=-\frac{\log(1-w_{n}/n)}{1-\delta }$ (B 6)	$\hat{\lambda }=\frac{\bar{x}}{1-\delta },\;\hat{r}=\frac{{\left(\bar{x}\right)}^{2}}{s_{x}^{2}-\bar{x}}$ (5.7)
*δ* = 0	$\hat{\lambda }=-\log(1-\hat{\alpha })=-\log\left(1-\frac{w_{n}}{n}\right)$ (B 2)	$\hat{\lambda }=\bar{x},\;\hat{r}=\frac{{\left(\bar{x}\right)}^{2}}{s_{x}^{2}-\bar{x}}$ (5.6)

## Data Availability

This article has no additional data.

## Appendix A. Technical results

Lemma A.1. *Let* *r*, *s* *be positive real numbers such that* *r* ≥ *s*. *Then*,

$$\lceil r\rceil -\lceil s\rceil =\left\{ \begin{array}{cc} \lceil r-s\rceil \; & or\;\\ \lceil r-s\rceil -1\; & \; \end{array}\right.$$

Proof of lemma A.1. We can decompose *r* = *m*_1_ + *a*_1_ and *s* = *m*_2_ + *a*_2_ with *m*_1_, *m*_2_ non-negative integers and 0 ≤ *a*_1_, *a*_2_ < 1 (note that *r* ≥ *s* implies that *m*_1_ − *m*_2_ is a non-negative integer). Then,

A 1 $$\lceil r\rceil -\lceil s\rceil =\left\{ \begin{array}{cc} (m_{1}+1)-(m_{2}+1)=m_{1}-m_{2}\; & \text{if }a_{1},a_{2}>0\;\\ (m_{1}+1)-m_{2}=m_{1}-m_{2}+1\; & \text{if }a_{1}>0,a_{2}=0\;\\ m_{1}-(m_{2}+1)=m_{1}-m_{2}-1\; & \text{if }a_{1}=0,a_{2}>0\;\\ m_{1}-m_{2}\; & \text{if }a_{1}=a_{2}=0\; \end{array}\right.$$

On the other hand, since 0 ≤ *a*_1_, *a*_2_ < 1, we have that −1 < *a*_1_ − *a*_2_ < 1 and therefore,

$$\lceil a_{1}-a_{2}\rceil =\left\{ \begin{array}{cc} 1\; & \text{if }a_{1}-a_{2}>0\;\\ 0\; & \text{otherwise, that is, if }a_{1}-a_{2}\leq 0,\; \end{array}\right.$$

and therefore,

A 2 $$\begin{array}{rl} \left\lceil r-s\right\rceil & =\lceil (m_{1}-m_{2})+(a_{1}-a_{2})\rceil =(m_{1}-m_{2})+\lceil a_{1}-a_{2}\rceil \\ & =\left\{ \begin{array}{cc} m_{1}-m_{2}+1\; & \text{if }a_{1}-a_{2}>0\;\\ m_{1}-m_{2}\; & \text{otherwise}.\; \end{array}\right. \end{array}$$

Then, from (A 1) and (A 2) it is trivial to check that

$$\left\{ \begin{array}{cc} \text{If }a_{1},a_{2}>0,a_{1}-a_{2}>0,\; & \lceil r\rceil -\lceil s\rceil =m_{1}-m_{2},\;\lceil r-s\rceil =m_{1}-m_{2}+1\;\\ \text{If }a_{1},a_{2}>0,a_{1}-a_{2}\leq 0,\; & \lceil r\rceil -\lceil s\rceil =m_{1}-m_{2},\;\lceil r-s\rceil =m_{1}-m_{2}\;\\ \text{If }a_{1}>0,a_{2}=0,\; & \lceil r\rceil -\lceil s\rceil =m_{1}-m_{2}+1,\;\lceil r-s\rceil =m_{1}-m_{2}+1\;\\ \text{If }a_{1}=0,a_{2}>0,\; & \lceil r\rceil -\lceil s\rceil =m_{1}-m_{2}-1,\;\lceil r-s\rceil =m_{1}-m_{2}\;\\ \text{If }a_{1}=a_{2}=0,\; & \lceil r\rceil -\lceil s\rceil =m_{1}-m_{2},\;\lceil r-s\rceil =m_{1}-m_{2}\; \end{array}\right.$$

concluding that in any case, ⌈r⌉−⌈s⌉ is either equal to or one smaller than ⌈r−s⌉, which concludes the proof. ▪

Proof of proposition 3.1. We first prove that for any *N* ≥ 1, (1.1)−(2.1)≥0. This is a consequence of the fact that

$$-\frac{1}{\lambda }\log(\beta +(1-\beta )\;e^{-\lambda N})<-\frac{1}{\lambda }\log(\beta ),$$

which is equivalent to (1 − *β*) e^−*λN*^ > 0 (that is trivially true), by the fact that the logarithm is an increasing function, and that the function *ceiling* is not decreasing.

(a) If for any *N* ≥ 1, we define
  $$\begin{array}{rl} r & =-\frac{1}{\lambda }\log(\beta )\;\text{and}\;s=-\frac{1}{\lambda }\log(\beta +(1-\beta )\;e^{-\lambda N}),\;\text{with}\;r>s.\;\text{Then},\\ r-s & =-\frac{1}{\lambda }\log(\beta )+\frac{1}{\lambda }\log(\beta +(1-\beta )\;e^{-\lambda N})=\frac{1}{\lambda }\log\left(\frac{\beta +(1-\beta )\;e^{-\lambda N}}{\beta }\right)\\ & =\frac{1}{\lambda }\log\left(1+\frac{1-\beta }{\beta }\;e^{-\lambda N}\right)>0 \end{array}$$
  and by lemma A.1,
  A 3 $$(1.1)-(2.1)=\left\lceil \frac{1}{\lambda }\log\left(1+\frac{1-\beta }{\beta }\;e^{-\lambda N}\right)\right\rceil \;\text{ or one smaller}.$$
  From which it follows immediately that $\left(1.1)-(2.1)\leq \lceil (1/\lambda )\log(1+(\left(1-\beta \right)/\beta )\;e^{-\lambda N})\right\rceil$.
(b) to see that (2.1) converges to (1.1) as *N* → +∞, we first check the limit
  $$\lim_{N\to \infty }-\frac{1}{\lambda }\log(\beta +(1-\beta )\;e^{-\lambda N})=-\frac{1}{\lambda }\log(\beta )$$
  which is a trivial check since e^−*λN*^ → 0. We now consider two different situations:
  (i) −(1/*λ*)log (*β*) is a positive integer, say *z*. So, ⌈z⌉=z and for *N* large enough, *z* − 1 < −(1/*λ*)log (*β* + (1 − *β*) e^−*λN*^) < *z* from which it follows that $\lceil -(1/\lambda )\log(\beta +(1-\beta )\;e^{-\lambda N})\rceil =z$.
  (ii) *z* < −(1/*λ*)log (*β*) < *z* + 1, with *z* a positive integer. Then, ⌈−(1/λ)log⁡(β)⌉=z+1 and for any *N* large enough, *z* < −(1/*λ*)log (*β* + (1 − *β*) e^−*λN*^) < −(1/*λ*)log (*β*) < *z* + 1, for which $\lceil -(1/\lambda )\log(\beta +(1-\beta )\;e^{-\lambda N})\rceil =z+1$.
  In both cases, (2.1)=(1.1) for *N* large enough, resulting in $\lim_{N\to +\infty }(2.1)=(1.1)$.
(c) Finally, if we set *λ* > 0 and *β* ∈ (0, 1), we introduce the function *f* by
  $$f\;(N)=\frac{1}{\lambda }\log\left(1+\frac{1-\beta }{\beta }\;e^{-\lambda N}\right)>0,$$
  then *f* is a monotonically decreasing function of *N*, since *f*′(*N*) = −((1 − *β*)/*β*) e^−*λN*^/(1 + ((1 − *β*)/*β*) e^−*λN*^) < 0, which converges to 0 as *N* → +∞. Since the function *ceiling* is nondecreasing, ⌈f (⋅)⌉ is a monotonically decreasing function of *N*, and converges to 1 as *N* → ∞. This fact, together with (A3) and that $\lim_{N\to +\infty }(2.1)=(1.1)$, necessarily imply that for *N* large enough,
  $$(1.1)-(1.2)=\left\lceil \frac{1}{\lambda }\log\left(1+\frac{1-\beta }{\beta }\;e^{-\lambda N}\right)\right\rceil -1,$$
  finishing the proof.

 ▪

## Appendix B. Estimation of the parameter *λ* in model A

When unknown, the parameter *λ* in model A, which is the mean value of the Poisson distribution that models the number of individuals of the target species in a (generic) sample, must be estimated. The procedure for doing this depends on whether the *false negative* rate *f*_*n*_ = *δ* can be considered to be =0 or >0.

**B.1. Case *δ* = 0**

Instead of estimating *λ*, we can equivalently estimate *α*, which is defined as *the probability that a sample contains at least one individual of the target species*, i.e.

$$\alpha =P(X>0)=1-e^{-\lambda }.$$

With this notation, *λ* can be obtained from *α* by *λ* = −log (1 − *α*) and to estimate the value of *λ*, fixed *N*, we could estimate *α* instead. Indeed, by its definition, *α* can be estimated by the proportion of samples that contain at least one individual of the target species, of those that have been taken from the area. That is, if we take *n* samples from the area *A*, then the estimate of *α* is

B 1 $$\hat{\alpha }=\frac{w_{n}}{n}$$

with *w*_*n*_ *the number of the* *n* *samples containing at least one individual of the target species*, and therefore the estimate of *λ* is

B 2 $$\text{model A},\;\delta =0\;:\;\;\hat{\lambda }=-\log(1-\hat{\alpha })=-\log\left(1-\frac{w_{n}}{n}\right)$$

Then, the estimated value of *n* can be obtained from the formula (2.1). In this way, we have reached a situation of ‘a snake biting its own tail’, that is, a vicious circle: we need the estimation of *α* to determine *n*, and at the same time we need a value of *n* to obtain an estimate of *α* by (B 1). The way to break it is to first estimate *α* by setting an arbitrary value of *n*, say *n*_0_, by (B 1), and then use the estimated value of *α* to get the *n* optimal.

Can we know, *a priori*, what value of *n*_0_ will give a ‘good’ (in a certain sense) estimate of *α*? The answer is yes, and it is based on the *confidence interval* of the estimate. Since $\hat{\alpha }$ is a proportion, its asymptotic confidence interval with a fixed confidence level of *γ* ∈ (0, 1) is

B 3 $$\hat{\alpha }\pm Z_{\left(1+\gamma \right)/2}\sqrt{\frac{\hat{\alpha }(1-\hat{\alpha })}{n_{0}}}$$

where *Z*_(1+*γ*)/2_ is obtained from the standard Normal distribution as the inverse of its distribution function at (1 + *γ*)/2. For example, if *γ* = 0.95, then (1 + *γ*)/2 = 0.975 and *Z*_0.975_ = 1.96. From (B 3) we can isolate *n*_0_ so that half the interval length, which is the error in estimating *α* by $\hat{\alpha }$, is no greater than a fixed error level ɛ > 0, in this way:

$$Z_{\left(1+\gamma \right)/2}\sqrt{\frac{\hat{\alpha }(1-\hat{\alpha })}{n_{0}}}\leq \varepsilon \;⟺\;Z_{\left(1+\gamma \right)/2}^{2}\frac{\hat{\alpha }(1-\hat{\alpha })}{n_{0}}\leq \varepsilon^{2}$$

and then, we use $\hat{\alpha }(1-\hat{\alpha })\leq 1/4$ (in fact, the function *f*(*x*) = *x* (1 − *x*) reaches its global maximum, which is 1/4, when *x* = 1/2). So, if we isolate *n*_0_ from the following inequality,

$$Z_{\left(1+\gamma \right)/2}^{2}\frac{1/4}{n_{0}}\leq \varepsilon^{2},$$

we make sure that what we wanted is fulfilled. Indeed, we make sure of it with

B 4 $$n_{0}\geq Z_{\left(1+\gamma \right)/2}^{2}\frac{1}{4\varepsilon^{2}}.$$

For example, if *γ* = 0.95 is the confidence level, and *ɛ* = 0.05 is the error level, from (B 4) we obtain that the number of samples to take from area *A* to estimate *α* by (B 1) with an error no greater that *ɛ* (saying this with a confidence of approximately *γ*), is

$$n_{0}\geq Z_{\left(1+\gamma \right)/2}^{2}\frac{1}{4\varepsilon^{2}}=1.96^{2}\frac{1}{4\times 0.05^{2}}=384.16.$$

So, *n*_0_ = 385 samples are enough to estimate *α* by the number of those samples that contain at least one individual of the target species ((B 1) with *n* = *n*_0_).

Note that *a posteriori*, the approximation (B 4) can be used if the estimated value of *α* obtained from *n*_0_ samples, $\hat{\alpha }$, verifies that $n_{0}\hat{\alpha }(1-\hat{\alpha })$ is large enough (say, greater than 20, which is a commonly accepted reference value for approximating the Binomial distribution by the Normal, although the approximation is better the larger the triple product is).

Remark B.1. What happens if the estimate of *α* that we find is zero? In other words: what if all the samples we draw do not contain individuals of the target species? Using the classical formula (B 3) leads to a dead end with a confidence interval reduced to 0. What then? Instead, we can try to find a *likelihood interval* for the estimate of *α* in the sense that a fixed ‘likelihood’ level *γ*, the probability of observing that none of the *n*_0_ samples have individuals of the target species is greater than 1 − *γ* (not too small):

$${\left(1-\alpha \right)}^{n_{0}}\geq 1-\gamma ,$$

which is equivalent to

B 5 $$n_{0}\log(1-\alpha )\geq \log(1-\gamma ).\;(B\;5)$$

The MacLaurin series for log (1 − *α*) is

$$\log(1-\alpha )=-\sum_{\ell =1}^{\infty }\frac{\alpha^{\ell }}{\ell }=-\alpha -\frac{\alpha^{2}}{2}-\frac{\alpha^{3}}{3}-\cdots$$

and therefore, using *α* to be close to zero (it must be, since its estimate turned out to be zero), we have that log (1 − *α*) ≈ −*α*, and replacing log (1 − *α*) with −*α* in the expression (B5), we can isolate *α* and get

$$\alpha \leq -\frac{\log(1-\gamma )}{n_{0}},$$

that is, the approximate *likelihood interval* for *α* with ‘likelihood’ level *γ* is

$$[0,-\frac{\log(1-\gamma )}{n_{0}}].$$

As a particular case, if *γ* = 0.95, the *likelihood interval* is

$$[0,-\frac{\log(0.05)}{n_{0}}]=[0,-\frac{-2.995732}{n_{0}}]\approx [0,\frac{3}{n_{0}}\;],$$

which is where the usual name of ‘rule of three’ comes from.

**B.2. Case *δ* > 0**

To estimate *λ* in scenario 2 (non-detection errors with probability *δ* > 0), we will use the parameter *α* defined now as *the probability that a sample contains at least one individual of the target species and we detect its presence*. With similar arguments to the proof of theorem 4.1, we can see that

$$\alpha =1-e^{-\lambda (1-\delta )}$$

and we can isolate *λ* and express it as a function of *α*, given that *δ* is known, by *λ* = −log (1 − *α*)/(1 − *δ*). Then, *α* can be estimated by the proportion of samples that have been taken from the area, for which the presence of at least one individual of the species has been detected. That is, if we take *n* samples from the area *A*, then the estimate of *α* is again given by the expression (B 1), but now *w*_*n*_ is *the number of the* *n* *samples in which we have detected the presence of the species*.

Analogously to the *δ* = 0 case, we first estimate *α* by setting an arbitrary value of *n*, say *n*_0_, and then obtain the estimate of *λ* analogously to (B 2) by

B 6 $$\text{model A},\;\delta >0\;:\;\;\hat{\lambda }=-\frac{\log(1-\hat{\alpha })}{1-\delta }=-\frac{\log(1-\frac{w_{n}}{n})}{1-\delta }$$

and then get the optimal value of *n* from formula (4.1).
